# Quality of Municipal Long-Term Care in Different Models of Care: A Cross-Sectional Study From Norway

**DOI:** 10.1177/11786329231185537

**Published:** 2023-07-17

**Authors:** Hanne Marie Rostad, Lisa Victoria Burrell, Marianne Sundlisæter Skinner, Ragnhild Hellesø, Maren Kristine Raknes Sogstad

**Affiliations:** 1 Center for Care Research East, Norwegian University of Science and Technology, Gjøvik, Norway; 2 Department of Public Health Science, University of Oslo, Oslo, Norway

**Keywords:** Health care delivery, long-term care, registry data, survey, quality indicators, health care, quality of care

## Abstract

The quality of care remains a critical concern for health systems around the globe, especially in an era of unprecedented financial challenges and rising demands. Previous research indicates large variation in several indicators of quality in the long-term care setting, highlighting the need for further investigation into the factors contributing to such disparities. As different ways of delivering long-term care services likely affect quality of care, the objectives of our study is to investigate (1) variation in structure, process and outcome quality between municipalities, and (2) to what extent variation in quality is associated with municipal models of care and structural characteristics. The study had a cross-sectional approach and we utilized data on the municipal level from 3 sources: (1) a survey for models of care (2) Statistics Norway for municipal structural characteristics and (3) the National Health Care Quality Indicator System. Descriptive statistics showed that the Norwegian long-term care sector performs better (measured as percentage or probability) on structure (85.53) and outcome (84.86) quality than process (37.85) quality. Hierarchical linear regressions indicated that municipal structural characteristics and model of care had very limited effect on the quality of long-term care. A deeper understanding of variation in service quality may be found at the micro level in healthcare workers’ day-to-day practice.

## Background

When healthcare systems focus on quality, a lot can be achieved: from financial savings, better preparedness for a large-scale emergency or medical event, and most importantly, saving patients’ lives.^1^ Globally, quality is recognized as a key factor in healthcare, and action is taken from involved constituencies – governments, health systems, citizens, patients and health workers – to achieve the goal of high-quality health service delivery at the front line.^1^

Several definitions of quality exist, but there are some established elements constituting quality in healthcare service; they should be effective, safe, person-centred, timely, equitable, integrated, available and efficient.^1^ Quality indicators, defined as ‘a quantitative measure that provides information about a variable that is difficult to measure directly’,^2^ are used worldwide to monitor, assess and report the quality of healthcare. These quality indicators often measure specific *structures* (eg, staff and patient ratios), *processes* (eg, assessment of nutritional status) and/or *outcomes* (eg, re-hospitalization).^3^

Previous research from Norway and other Nordic countries indicates large variation in several indicators of quality in the long-term care setting, both between municipalities and between long-term care settings (eg, nursing homes and home healthcare services). Examples are differences between municipalities in the provision of day activities for people with dementia, number of days discharge-ready patients remain hospitalized and availability of nursing home and home healthcare services.^4^ Other examples include differences between nursing homes, or differences between nursing homes and home healthcare services, in patients’ oral health status,^5^ nutritional follow-up,^6-8^ medication-related problems^9^ and in the quality of drug prescribing.^10^ Occasionally, this is picked up by the media, highlighting anecdotes of failure in some municipalities and their long-term care providers concerning the quality of long-term care. This sparks debates concerning inter-municipal differences and to what extent quality of long-term care services is determined by the municipality you live in and get your long-term services from.

Looking ahead, the increase in the proportion of older adults with reduced physical or cognitive function triggers a growing demand for long-term care in Europe, now and for years to come.^11^ Scandinavian countries’ spending on long-term care is higher than most EU countries (around 3.5% of GDP compared to an average of, 1.5%)^12^ and Norway has achieved substantial progress in shifting non-acute care away from hospitals to municipal based settings, such as long-term care services,^13^ making Norway an interesting case for studies about long-term care.

Norway has a municipality-based and publicly funded long-term care, serving 371 319 people in 2020, which constitutes 6.9% of Norway’s population.^14^ There are national and local guidelines, rules and regulations for long-term care services. At the same time, Norwegian municipalities have autonomy and flexibility in designing their long-term care services, resulting in different models of care provision, distribution and deliverance. In previous work, we have identified and characterized 4 *models of care* in long-term care services in Norway which differed with regard to their prioritization of 4 core modes of service delivery: *Specialized municipal service, Assistive technology, Health promotion and Activity* and *Planning and coordination of care*.^15^ These long-term care models can be an indication of the municipalities’ strategic priorities, and we put forward that such strategic priorities may affect municipalities’ care provision and the quality of the care they deliver.

We also recognize that Norwegian municipalities vary significantly in size, demography and economy. We need more knowledge on how such structural characteristics may impact quality of the long-term care services. Therefore, the objectives of the current article, using Norway as a case, is to investigate (1) variation in structure, process and outcome quality between municipalities, and (2) to what extent variation is associated with municipal models of care and structural characteristics.

## Materials and Methods

The present study utilized data on the municipal level from 3 sources: (1) a survey used to identify and characterize models of care, (2) Statistics Norway, where we retrieved data on municipal structural characteristics and (3) the National Health Care Quality Indicator System database, where we retrieved data on quality of long-term care.^16^ A cross-sectional approach was used for this study, and all data were from 2019.

### The survey: Models of care

We conducted a survey to map municipalities’ provision of long-term care services for adults (ie, individuals over the age of 18). The majority of the questions concerned the traditional long-term care services: home healthcare and nursing homes. All Norwegian municipalities were invited to participate. Of 422 municipalities, 277 (65.7%) responded. Through a hierarchical cluster analysis, we clustered the 277 municipalities into 4 models of care based on 4 modes of service delivery, with scores ranging from 0 to 100. *Specialized municipal service, Assistive technology, Health promotion and Activity* and *Planning and coordination of care*.

More information about the questionnaire, recruitment of respondents and the process of identifying and describing our 4 models of care, can be found in other publications.^15^ The variable for models of care is used as a *predictor variable* and has 4 categories:

• Care model 1
The municipalities belonging to this model had low to moderate scores on all the modes of service delivery. The highest (but moderate) mean score was for *Health promotion and Activity*. Most of the municipalities (121 out of 277) fitted this model.
• Care model 2
This group of municipalities had highest scores on *Planning and coordination of care* followed by *Health promotion and Activity. Specialized municipal services* and *Assistive technology* in service provision had moderate and lower scores for this cluster. A total of 105 municipalities corresponded to this model.
• Care model 3
These municipalities had highest scores on *Assistive technology* and *Health promotion and Activity* and lowest scores on *Planning and coordination of care* and *Specialized municipal services*. A total of 35 municipalities matched this model.
• Care model 4
The municipalities belonging to this model had high scores on all modes of service delivery. The highest scores for this cluster were on *Health promotion and* Activity, and *Planning and coordination of care* and this cluster had the highest degree of *Specialized municipal services* compared to the other clusters. They also gave an above-average priority to *Assistive technology* in service provision. Only 16 municipalities corresponded to this model.

### Statistics Norway: Municipal structural characteristics

Statistic Norway’s database includes statistics on several topics, such as population data and municipal characteristics and activities. All Norwegian municipalities report to Statistics Norway annually, and the information is publicly available on Statistics Norway’s website.

The following are used as *control variables* in our analyses:

- Population size. A continuous variable of the number of inhabitants in the municipalities.^17^
- Centrality. A categorical variable with 3 categories: least central, central and most central. Statistics Norway’s centrality index is based on the travel time to workplaces and service functions (eg, post office and bank).^18^
- The proportion of older adults. A continuous variable for the percentage of the municipality’s inhabitants aged ⩾80.^19^
- Municipal income. Measured as ‘unrestricted revenues per capita’, which is a continuous variable for how much income the municipalities have at their disposal after covering the fixed costs, indicating the municipalities’ financial leeway.^20^

### The Health Care Quality Indicator System database: Structure, process and outcome quality

Quality is a theoretical concept that can encompass different aspects depending on the exact definition and the context of measurement, making it difficult to measure directly.^21^ In this paper, the conceptualization of quality is made in the particular setting of long-term care, using indicators that reflect the Norwegian health authority’s quality goals for this particular setting.

The Norwegian Directorate of Health, under the Ministry of Health and Care Services, is responsible for the Quality Indicator system. The Directorate develops, disseminates and maintains the quality indicators and collects data from the municipalities to assess, monitor and report quality and variation in quality between municipalities. The Norwegian National Quality Indicator system currently comprises more than 180 indicators, mostly process indicators for specialist healthcare services. For municipal health and care services, there are 24 indicators which include indicators concerning home healthcare, nursing home care, practical aid, transport services and more.^22^ Statistics for most of the indicators are open and available for public use.

We chose to include the 7 openly available indicators that measure aspects of the quality of nursing home and home health care services (Table 1). All 7 indicators are measured as percentage or probability, thus ranging from 0 to 100. These 7 quality indicators include both structure, process and outcome indicators, and were grouped accordingly using the ‘combine’ command in R which takes the variables’ values and concatenate them (eg, for ‘Structure’, 3 values were concatenated). We present descriptive results for the single indicators, but the *outcome variables* in our hierarchical linear regression models were composite indictors of structure, process and outcome quality.

**Table 1.** Composite indicators of structure, process and outcome quality.

Composite indicator	Quality indicator constituting the composite indicator	Measured as
Structure	Full-time equivalents with vocational training	The percentage of full-time equivalents of personnel with relevant vocational training, in total.
	Private room with bathroom, nursing home	The percentage of nursing home places that are private rooms with private bathroom and WC.
	Sick leave among personnel^a^	The percentage without absence due to illness among staff in the health and care services, in total.
Process	Nursing home residents assessed by dental health personnel	The percentage of residents on long-term stays in a nursing home that has been assessed by dental health personnel during the last 12 mo.
	Nutritional follow-up, home-dwelling	The percentage of homecare recipients, aged 67 and older, who have had their nutritional status assessed during the last 12 mo.
	Nutritional follow-up, nursing home residents	The percentage of nursing home residents, aged 67 and older, who have had their nutritional status assessed during the last 12 mo.
Outcome	Re-admissions to hospital among older adults^b^	The probability of no re-admission of older adults, aged 67 and older, within 30 d after discharge

a The original statistic is ‘The percentage of absence due to illness among staff in the health and care services, in total’. The scale was reversed in our analyses, showing the percentage without absence in order to meaningfully interpret the result of the structure quality group.

b The original statistic is ‘The probability of re-admission of older adults, aged 67 and older, within 30 days after discharge’. The scale was reversed in our analyses, showing the probability of no re-admission of older adults, aged 67 and older, within 30 days after discharge.

All variables composing the group now signals a positive change as the estimate increases.

### Data analysis

First, we used descriptive statistics to describe our variables through frequencies and measures of centrality and dispersion. To answer our research question, we used a hierarchical linear regression with 2 steps. In step 1, we included the municipal structural characteristics that we want to hold constant: population size, centrality, the proportion of older adults and municipal income. In step 2, we added municipal models of care in order to investigate their potential association with the quality indicators when controlling for structural characteristics of municipalities.

The data analyses were performed using the lm function in R. Exact *P*-values are reported rather than labelling our results as ‘statistically (in)significant’, as passing an arbitrary threshold such as *P* < .05 does not mean or imply that an association is highly probable, real, true or important.^23^ Missing data is handled with ‘half rule’ where only participants with valid observations on at least half of the items were included in the scale scores.^24^

### Ethics

Data from Statistics Norway and The Norwegian National Quality Indicator system are publicly available. The procedure of the survey study was assessed by the Data Protection Authority within the Norwegian Centre for Research Data (from 2022 ‘Sikt - Norwegian Agency for Shared Services in Education and Research’) who concluded that the processing of personal data was in accordance with privacy legislation (reference no. 847216). The municipalities received written information about the study and consented to participate by completing the survey.

## Results

In total, 276 municipalities were included in the analyses. Originally, 277 municipalities had answered our survey, but one municipality did not report on the 7 included quality indicators in 2019. Our sample varied in terms of municipalities’ geographical placements (5 regions: North, Mid, West, South and East) and structural characteristics, and is a good representation of the diverse conditions Norwegian municipalities operate under when designing their long-term services.

Descriptive information on municipality characteristics is presented in Table 2. Just under half of the municipalities in our sample belonged to Care model 1. These municipalities were less central, had fewer inhabitants and more unrestricted revenues, while municipalities in Care model 4 were more central, had more inhabitants and less unrestricted revenues.

**Table 2.** Characteristics of the municipalities, in total and for the different care models separately.

		Care model			
	Total	1	2	3	4
	N = 276	N = 121	N = 105	N = 34	N = 16
Population size, mean (SD)	14 523.17 (47 564.06)	6051.93 (7442.21)	19 412.53 (71 439.34)	14 133.44 (16 021.24)	47 328.62 (56 472.26)
Centrality, n (%)					
Least central	148 (53.62)	75 (61.98)	55 (52.38)	15 (44.12)	3 (18.75)
Central	106 (38.41)	42 (34.71)	40 (38.10)	17 (50.00)	7 (43.75)
Most central	22 (7.97)	4 (3.31)	10 (9.52)	2 (5.88)	6 (37.50)
Proportion of older adults aged ⩾80, mean (SD)	5.22 (1.35)	5.22 (1.37)	5.32 (1.32)	5.48 (1.23)	4.05 (1.10)
Unrestricted revenues per capita (NOK), mean (SD)	62 604.77 (12 713.87)	65 249.02 (15 437.31)	61 478.84 (9855.25)	59 754.68 (9790.04)	56 053.00 (6574.31)
Quality, mean (SD)					
Structure quality	85.53 (7.02)	84.81 (8.94)	85.93 (5.30)	87.11 (3.90)	84.95 (4.95)
Full-time equivalents with vocational training	77.77 (5.71)	78.30 (5.90)	77.66 (5.55)	77.10 (5.90)	75.94 (4.70)
Private room with bathroom, nursing home	90.04 (19.29)	87.38 (24.40)	91.25 (14.78)	95.32 (8.73)	91.02 (15.64)
Sick leave among personnel	89.01 (2.57)	88.99 (2.68)	89.03 (2.61)	89.37 (2.43)	88.20 (1.47)
Process quality	37.85 (21.45)	38.01 (24.49)	35.47 (19.76)	44.34 (17.79)	37.28 (12.79)
Nursing home residents assessed by dental health personnel	49.09 (27.81)	51.05 (30.04)	44.80 (28.09)	56.85 (21.22)	47.72 (16.94)
Nutritional follow-up, home dwelling	18.98 (17.07)	18.42 (18.21)	18.15 (17.17)	22.64 (14.30)	19.83 (14.93)
Nutritional follow-up, nursing home residents	44.07 (28.19)	42.55 (31.46)	42.73 (26.75)	52.63 (23.98)	44.14 (19.04)
Outcome quality	84.86 (1.11)	85.04 (1.06)	84.83 (0.93)	84.56 (1.61)	84.45 (1.01)
Overall quality	67.17 (10.09)	67.25 (11.35)	66.44 (9.43)	69.67 (7.83)	66.02 (8.36)

**Figure 1.**
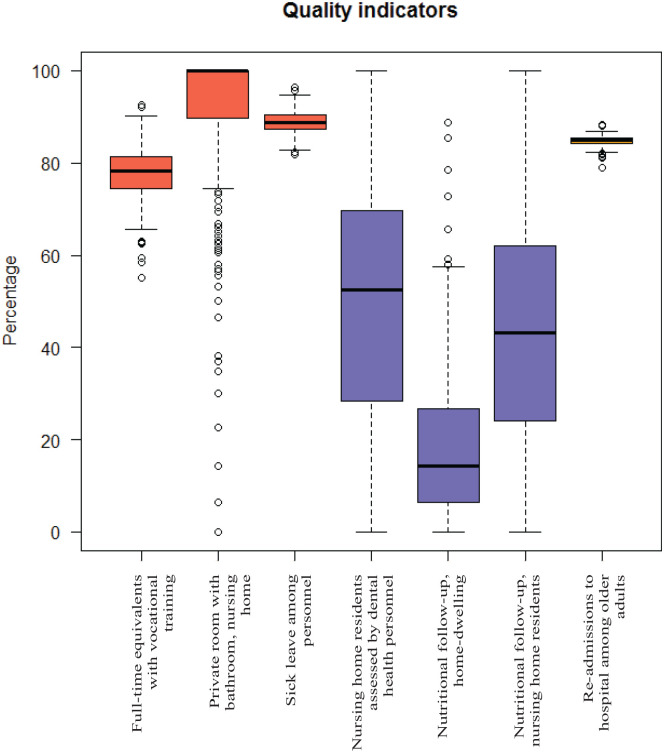
Box plots for all quality indicators, grouped by colour into structure (red), process (blue) and outcome (orange) quality.

Table 2 also presents the mean quality of all municipalities combined and for the different care models separately. Municipalities in Care model 3 had the highest process quality, while municipalities in Care model 2 had the lowest process quality. The centrality and dispersion for each quality indicator is visualized in Figure 1, showing the values for structure and outcome quality to be higher than those for process quality. Also, process quality varied a lot between municipalities, whilst there was less variation between the municipalities in structure quality and hardly any for outcome quality.

### Hierarchical linear regression models

Results from the hierarchical regression analyses are presented in Table 3.

**Table 3.** Hierarchical regression analyses for structure, process and outcome quality.

	Structure		Process		Outcome	
	Estimate (95% CI)	*P*	Estimate (95% CI)	*P*	Estimate (95% CI)	*P*
*Step 1 – Structural characteristics*						
Centrality						
Least central	ref.		ref.		ref.	
Central	1.25 (−1.11, 3.61)	.297	−8.93 (−16.42, −1.43)	.020	0.001 (−0.39, 0.39)	.994
Most central	1.67 (−2.39, 5.73)	.420	−16.05 (−28.52, −3.59)	.012	−0.46 (−1.11, 0.18)	.157
Population size	−0.01 (−0.03, 0.01)	.304	0.03 (−0.03, 0.09)	.303	−0.002 (−0.005, 0.002)	.338
Unrestricted revenues (per 10 000 NOK)	0.71 (−0.11, 1.53)	.088	−2.50 (−5.21, 0.21)	.070	0.12 (−0.09, 0.34)	.263
Proportion of older adults aged ⩾80	−0.07 (−0.86, 0.71)	.852	−2.41 (−5.05, 0.22)	.073	0.06 (−0.07, 0.20)	.356
*Step 2 – Structural characteristics + Models of care*						
Centrality						
Least central	ref.		ref.		ref.	
Central	0.99 (−1.38, 3.36)	.412	−9.60 (−17.14, −2.07)	.013	0.03 (−0.35, 0.42)	.864
Most central	1.38 (−2.74, 5.50)	.509	−16.12 (−28.80, −3.43)	.013	−0.40 (−1.05, 0.25)	.226
Population size	−0.01 (−0.03, 0.01)	.238	0.03 (−0.03, 0.09)	.283	−0.001 (−0.004, 0.002)	.422
Unrestricted revenues (per 10 000 NOK)	0.84 (0.02, 1.67)	.045	−2.43 (−5.15, 0.29)	.080	0.09 (−0.12, 0.31)	.398
Proportion of older adults aged ⩾80	−0.26 (−1.07, 0.55)	.525	−2.72 (−5.43, −0.01)	.049	0.09 (−0.05, 0.23)	.208
Models of care						
1	ref.		ref.		ref.	
2	1.51 (−0.38, 3.40)	.118	−2.02 (−8.22, 4.17)	.521	−0.18 (−0.47, 0.12)	.240
3	2.75 (−0.00, 5.50)	.050	7.67 (−1.01, 16.34)	.083	−0.46 (−0.89, −0.03)	.037
4	0.55 (−3.33, 4.42)	.782	−0.87 (−13.50, 11.77)	.893	−0.26 (−0.85, 0.34)	.399

Step 1 of the analyses included the variables we wanted to control for, that is, the municipal structural characteristics. Associations between centrality and process quality showed that more central municipalities have lower process quality. Compared to the least central municipalities, central municipalities scored, on average, 8.93 percentage points lower on process quality, while the most central municipalities scored 16.05 percentage points lower.

There was also a tendency for municipalities with greater unrestricted revenues to have slightly higher structure quality, but somewhat lower process quality, and for municipalities with a greater proportion of older adults to have a slightly lower process quality. Note, however, that the estimates related to unrestricted revenues and proportion of older adults are small.

Step 2 of the analyses included the municipalities’ model of care, which did not substantially change the associations between municipalities’ structural characteristics and quality. There were still small to moderate associations between centrality and process quality, unrestricted revenues and structure and process quality and the proportion of older adults and process quality. Furthermore, results showed that municipalities in Care model 3 had lower outcome quality, but a tendency towards higher structure and process quality. Note again that the estimates were small to moderate.

## Discussion

The current paper studied quality of long-term care in Norway and sought to determine whether variation in quality was associated with municipal care models and structural characteristics.

Overall quality score for Norwegian municipalities was 67.17%, and scores were higher for the structure (85.53%) and outcome measures (84.86%), and substantially lower for the process measures (37.85%). However, it is challenging to indicate whether our estimated values represent good or bad quality of care since the quality indicators used in this paper provide a measurement concept, but lack an appraisal concept, that is, a description of how the measure is expected to be used to judge quality.^21^ The Norwegian government has not established a reference point constituting adequate quality. However, our results indicate that the Norwegian long-term care sector performs better on the single indicators and composite measures of structure and outcome quality compared to the single indicators and composite measure of process quality.

As visualized in Figure 1, the quality indicators differed between municipalities: process quality varied greatly, structure quality varied slightly and outcome quality varied minimally. Further, we wanted to study how this variation could be explained by the 4 different municipal models of care while controlling for municipal structural characteristics. We put forward that different ways of delivering long-term care services would affect quality of care, but our results indicate that the model of care had very limited influence on quality.

The lacking influence of the models of care on quality can be due to different reasons. Firstly, the care models are highly correlated with the structural characteristics of the municipalities, such as unrestricted revenues, population size and centrality. For example, the municipalities in Care model 1 are small and rural, while the municipalities in Care model 4 are large and central. Consequently, the possible effects of care models may be eliminated when controlling for the municipal characteristics. Secondly, our quality indicators are aggregated from different service units in the municipalities, potentially hiding existing variation. In general, the true extent of variation is masked when outcomes are aggregated over larger geographic areas.^25^ Thirdly, the structure and outcome quality indicators are highly affected by factors such as case-mix, healthcare provider, institutional and organizational characteristics and the local labour market.^22^ For process indicators, the tasks and procedures are heavily influenced by institutional culture and routines, individual professional judgement and/or personalization of care. Our results thus indicate that a better understanding of variation in service quality requires further studies on the micro level. Lastly, our modes of service delivery, and thus our care models, may involve factors that have limited influence on our quality indicators. For example, having a high level of specialization in nursing home and home healthcare does not necessarily translate into performance on ‘nutritional follow-up’. If it does, we do not know ‘how’; if being highly specialized leads to more frequent nutritional follow-up or less, or if it would be in the same direction in every municipality. Additionally, our modes of service delivery and care models are not exhaustive, and are missing more dimensions that, given previous research and policy priorities, would be interesting to explore, such as person-centred care delivery,^26,27^ work-engagement among staff,^27^ effective training and professional development organizational culture and leadership.^28^

Previous research has been done to identify and describe organizational home care models across Europe,^29^ as a start to investigate the association between clients’ health outcomes and the home care models,^30^ however due to large variation between countries the outcome has so far only been linked to structural characteristics of the countries health care systems.^31^ There are also some research into benefits of integrated care models in improving the quality of life and functionality of people with multimorbidity and frailty in long-term care facilities,^32^ and some studies looking into relationship between different structural characteristics and quality indicators in long-term care.^33,34^ Differences between the health care systems makes it difficult to compare these to findings from our study. However, the overarching challenges and external drivers of change in long-term care are similar in many countries,^35^ and quality of care is the utmost priority for everyone involved in healthcare, around the globe. Thus, our contribution gives novel insight into how Norway per 2019 has responded to the challenges facing long-term care (our models of care) and the effect it has had on quality, which may provide other countries with ideas and solutions.

Our study illustrates that attributing quality differences to municipal model of long-term care is objectionable. However, this does not mean that the endeavour was pointless. Our contribution is informative, as it identifies whether some ways of delivering long-term care yield better results, in terms of quality output, compared to others. This helps to shed light on the long-term care sector’s struggles with disquieting local inequality and is key information for policymakers who govern long-term care services to assure high quality care delivery. It is also important information for municipal leaders, long-term care managers and individual healthcare providers.

### Limitations

Quality is both a subjective value-based issue and something which is defined by objective measures. It is a limitation that this study solely includes objective measures which are defined by experts through external criteria. The quality indicators are created from an intended goal or objective of the Norwegian health authorities but may not be in line with what health care providers or patients find important or meaningful in terms of quality of long-term care.

Data coverage varied between municipalities. It is also likely that data quality varied between and within municipalities and differed from indicator to indicator. We used data from 2019, a year when many municipalities were preparing for municipal mergers. The Norwegian Directorate of Health recognizes that this have impeded the reporting capacity of many municipalities, resulting in underreporting.

## Conclusion

The Norwegian long-term care sector performs better on structure and outcome quality than process quality. Furthermore, municipal characteristics and model of care had very limited effect on the quality of long-term care. A deeper understanding of variation in service quality may be found at the micro level in healthcare workers’ day-to-day practice.
